# Supplementary material for: Trametinib Induces the Stabilization of a Dual GNAQ p.Gly48Leu- and FGFR4 p.Cys172Gly-Mutated Uveal Melanoma. The Role of Molecular Modelling in Personalized Oncology
Source: Int J Mol Sci. 2020 Oct 28;21(21):8021. doi: 10.3390/ijms21218021 (PMC7662249; doi:10.3390/ijms21218021)
Supplement: Supplementary file 1 [file ijms-21-08021-s001.pdf]

Trametinib Induces the Stabilization of A Dual *GNAQ* p.Gly48Leu- and *FGFR4* p.Cys172Gly-Mutated Uveal Melanoma. The Role of Molecular Modelling in Personalized Oncology

Fanny S. Krebs [1], Camille Gérard [2], Alexandre Wicky [2], Veronica Aedo-Lopez [3], Edoardo Missiaglia [4,5], Bettina Bisig [5], Mounir Trimech [5], Olivier Michielin [2,3,4], Krisztian Homicsko [2,4,6] and Vincent Zoete [1,4,*]

Appendix A

FoldX procedure

FoldX is a well-known software for predicting changes in free energy of folding caused by mutations. It permits a rapid estimation of the effect of mutations on the stability of proteins and nucleic acids. Its predictive efficiency has been trained on a large set of mutants covering most of the existing structural environment. The energy terms used in FoldX estimation calculations have been weighted using empirical data obtained from protein engineering experiments [http://foldxsuite.crg.eu/products]. Potapov et al. [1] demonstrated that FoldX_3.0 shown the best correlation (r = 0.96) between experimental and average predicted change in protein stability values (ΔΔG), compare to other software like Rosetta and I-Mutant_2.0.
To predict the potential impact of a mutation in a structure, the FoldX command line *PositionScan* was used. In this procedure, the selected residue is first mutated to alanine and all neighbors residues are identified. Then, FoldX "mutates" the wild-type residue (selected position) to itself. Then, the neighbor residues are "mutated" to themselves; this is followed each time by the "mutation" of the wild-type selected position to itself. This process guarantees that the virtual *real* mutation by FoldX will potentially move only residues that were moved in the wild-type reference. Finally, the selected position is mutated into the desired residue.
Command line example:

```
FoldX --command=PositionScan --pdb=PDB_ID --positions=GA48L
```

In this example, FoldX will mutate Gly48 from the chain A into Leucine.
The output result corresponds to the ΔΔG in kcal/mol between the wild type and the mutant free energy structures ($\Delta\Delta G = \Delta G_{Mutant} - \Delta G_{Wild\text{-}Type}$).

Experimental structure selection

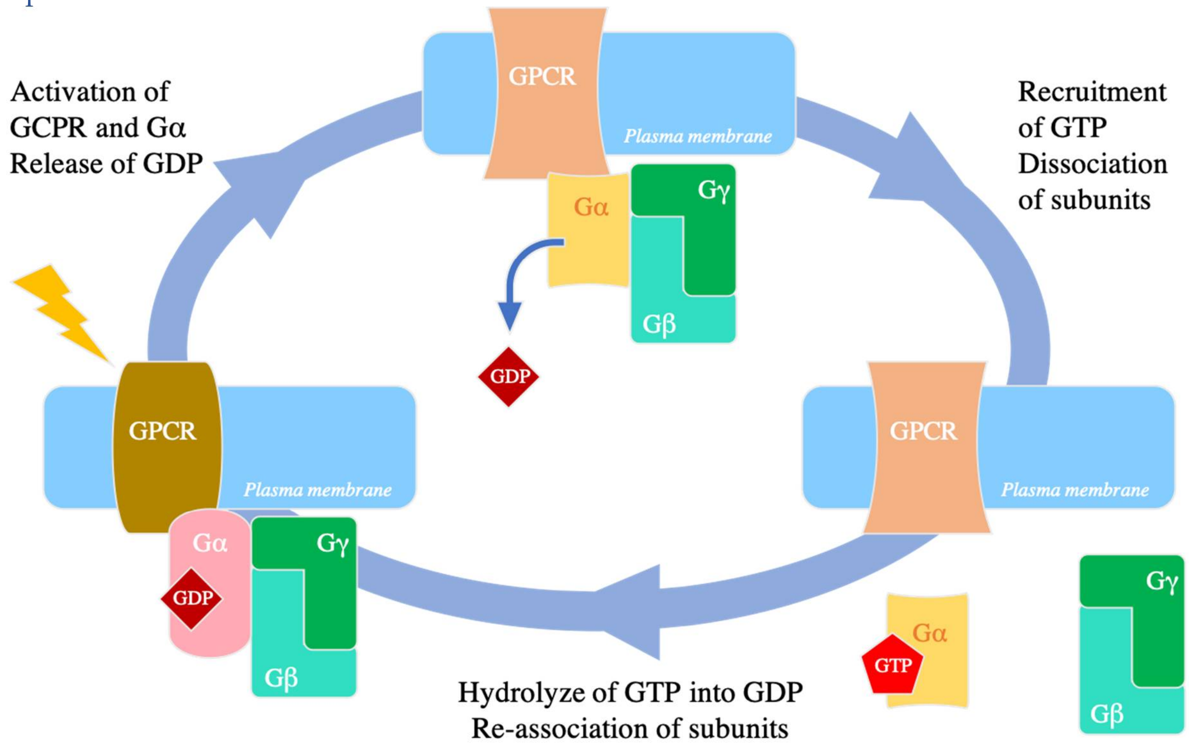

Figure S1. GCPRs and G protein subunits activation cycle.

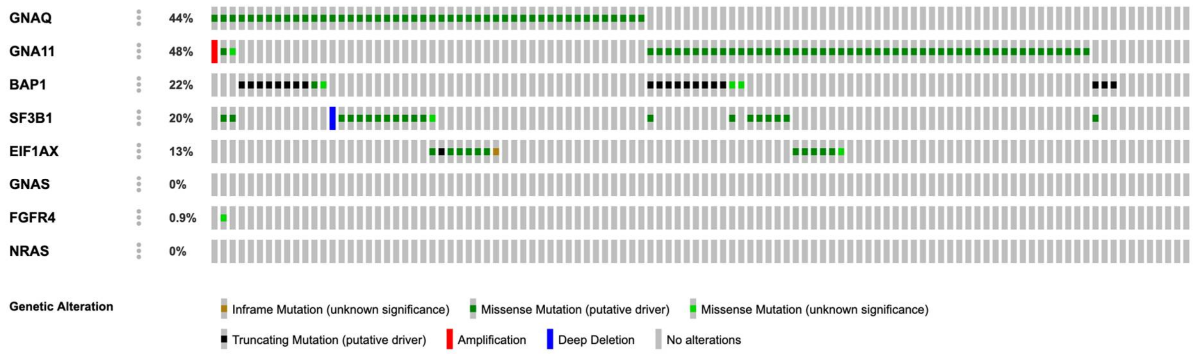

Figure S2. cBioportal oncoprint of the five mostly mutated genes (*GNAQ, GNAQ11, BAP1, SF3B1, EIF1AX*), plus mutated genes detected in our patient case, NGS results detected in 108 uveal melanoma patients of two cohorts (n = 28 [1] and n = 80 [2]) [3,4]

Table S1. Sequence identity of FGFR4 with FGFR1,-2 and -3.

	Sequence identity with FGFR4
FGFR1	57%
FGFR2	58%
FGFR3	61%

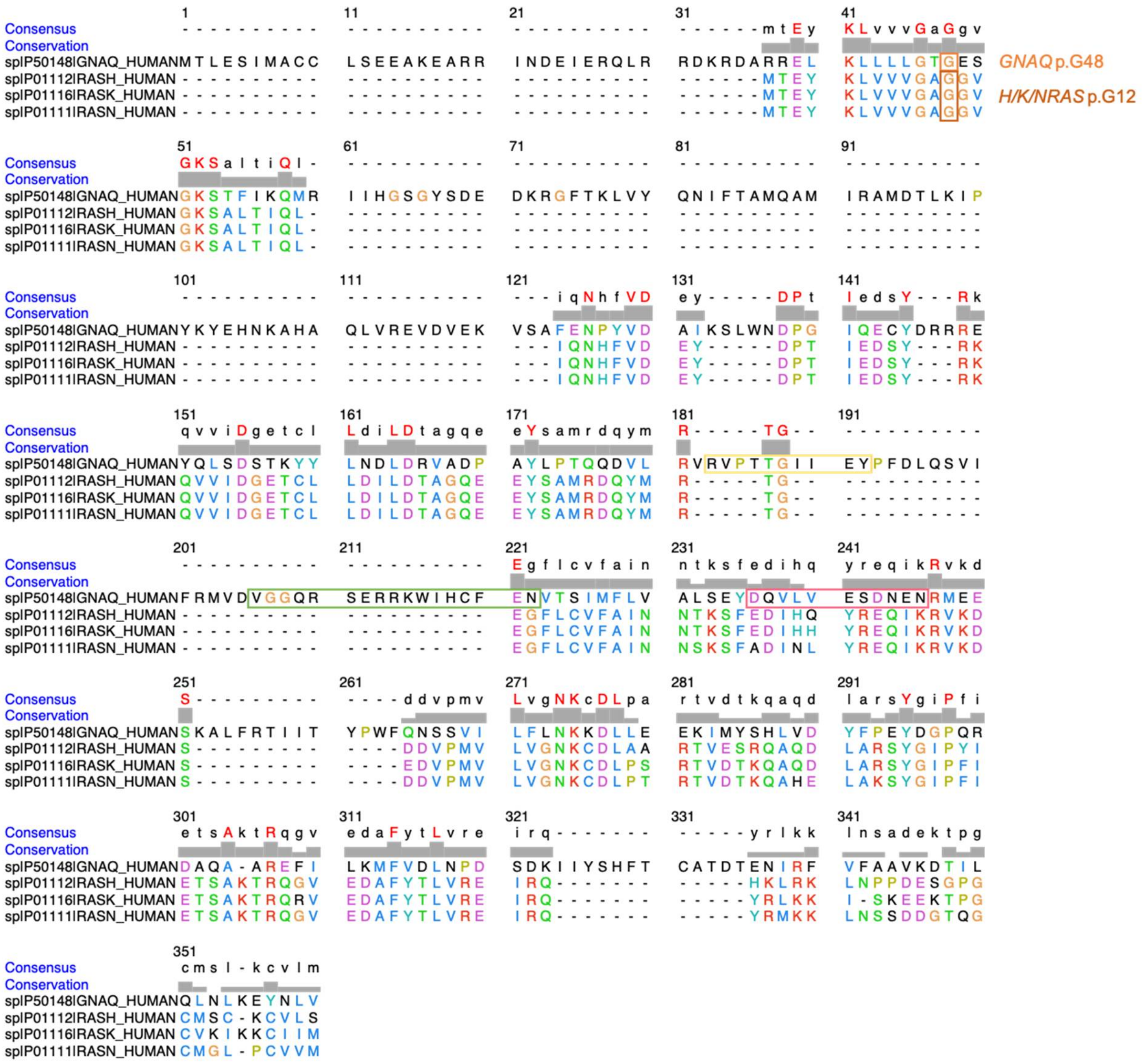

Figure S3. Sequence alignment of human *H*/*K*/*NRAS* and human *GNAQ*. Interestingly, *GNAQ* p.G48 corresponds to *H*/*K*/*NRAS* p.G12 which is a hotspot in RAS. The yellow, green and pink rectangles indicate the switch regions I, II and III in *GNAQ*, respectively.

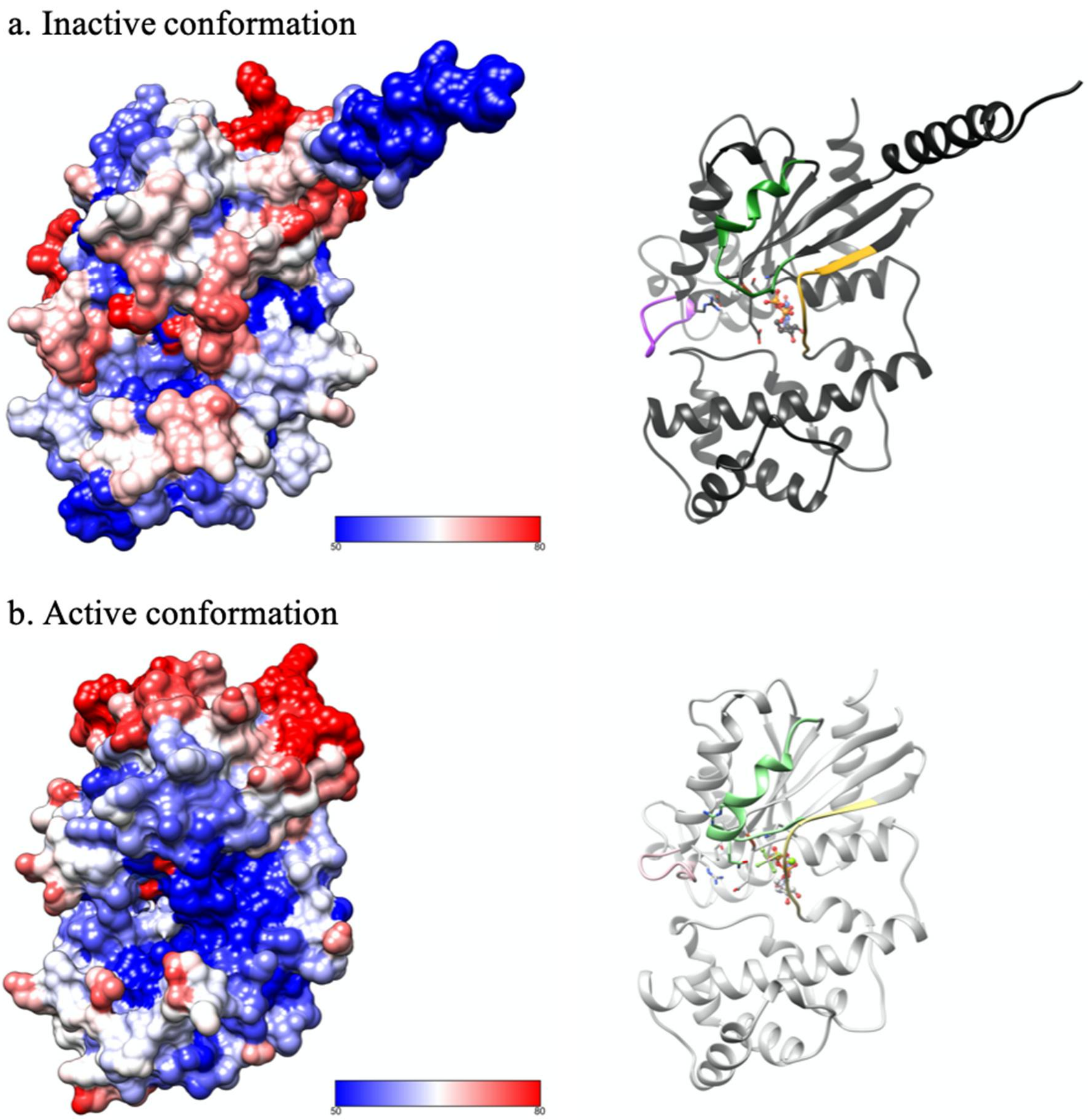

Figure S4. B-factor representation of inactive and active structures. (**a**). The active conformation was obtained as a co-crystal with GDP, AlF_4 and Mg^{2+} (PDB ID 3ohm); (**b**). The inactive conformation as a co-crystal with GDP (PDB ID 3ah8). The gradient corresponding to the B-factor values goes from blue to red, for rigid and flexible regions respectively. GDP and AlF_4 are shown in ball&stick and the Mg^{2+} in sphere representations, respectively. SW-I (183-192), SW-II (206-222) and SW-III (236-246) are respectively colored in gold, green and salmon.

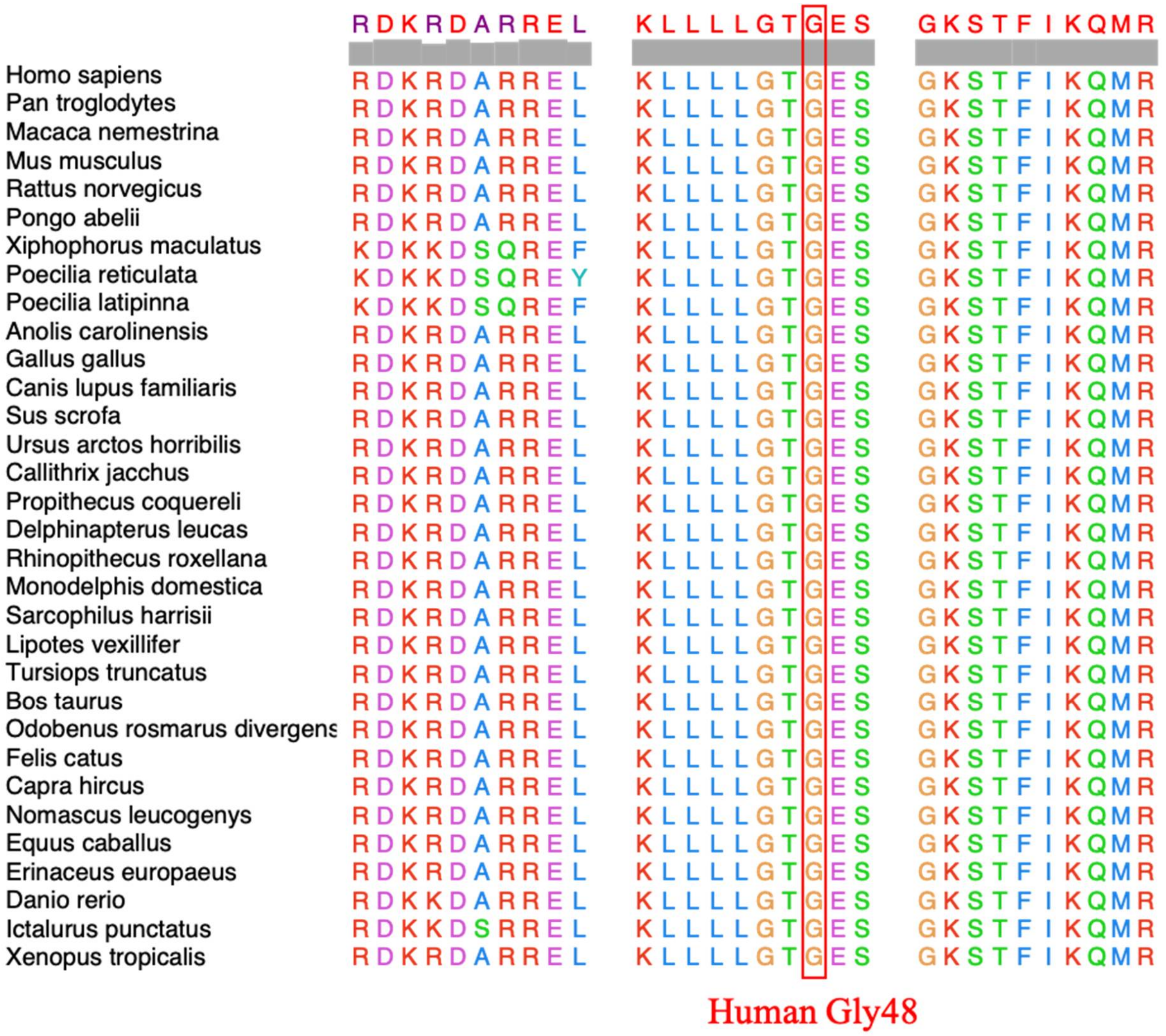

Figure S5. Sequence alignment of *GNAQ* orthologs centred on Human Gly48.

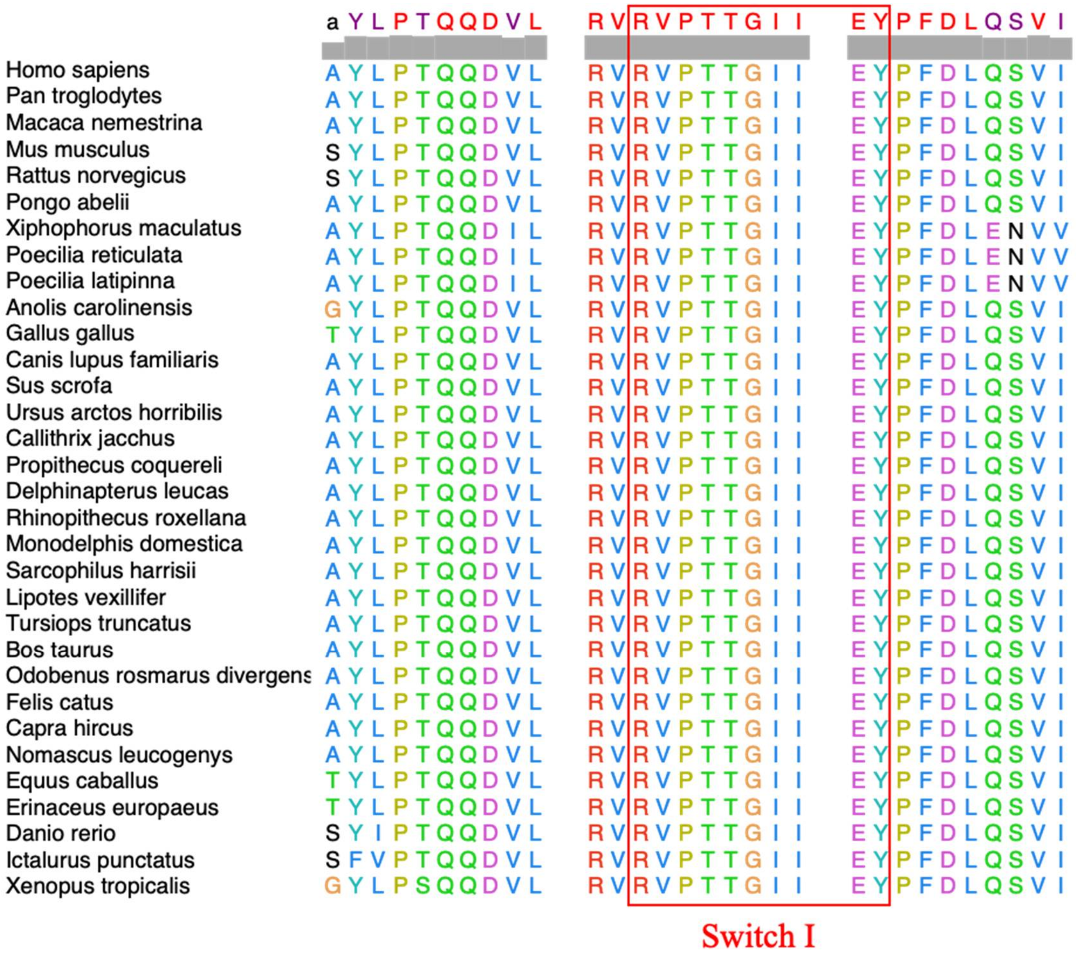

Figure S6. Sequence alignment of *GNAQ* orthologs centred on the Switch I loop (residues 183-192 based on human sequence).

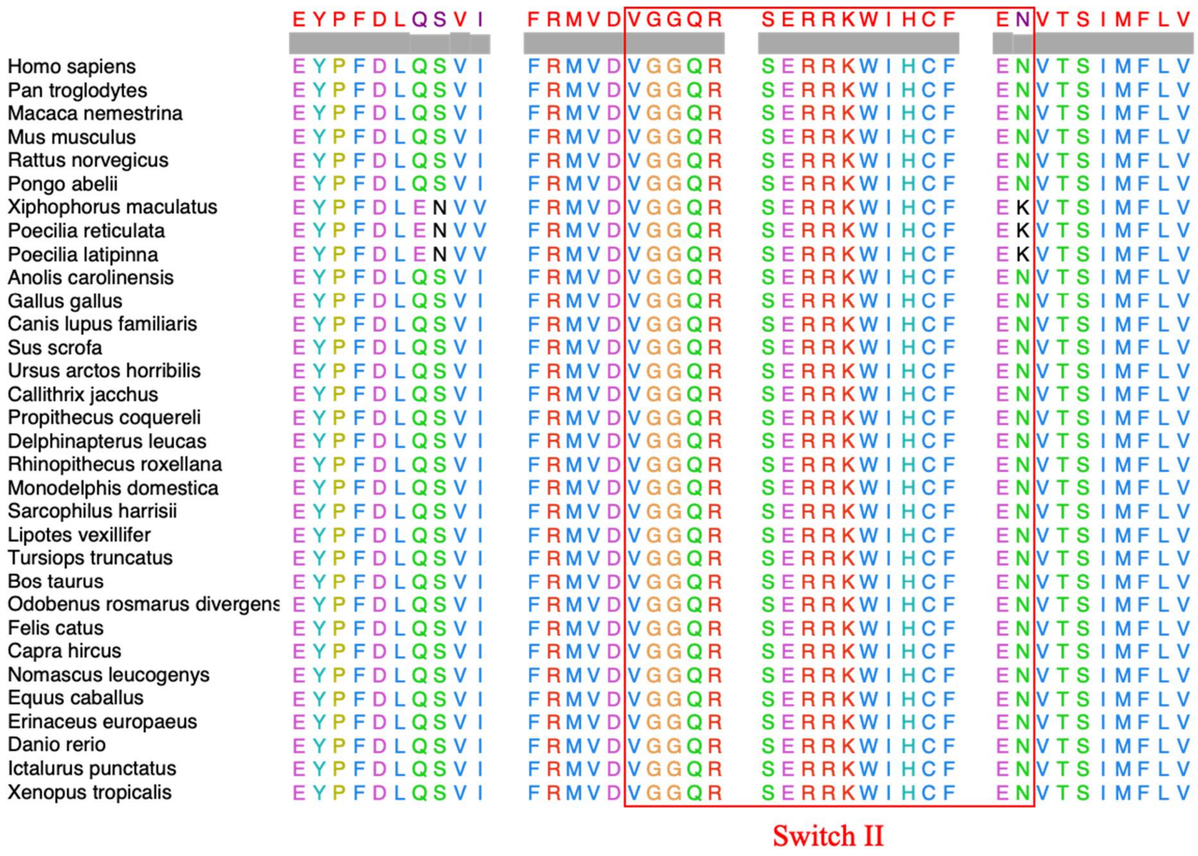

Figure S7. Sequence alignment of *GNAQ* orthologs centred on the Switch II loop (206-222 based on human sequence)

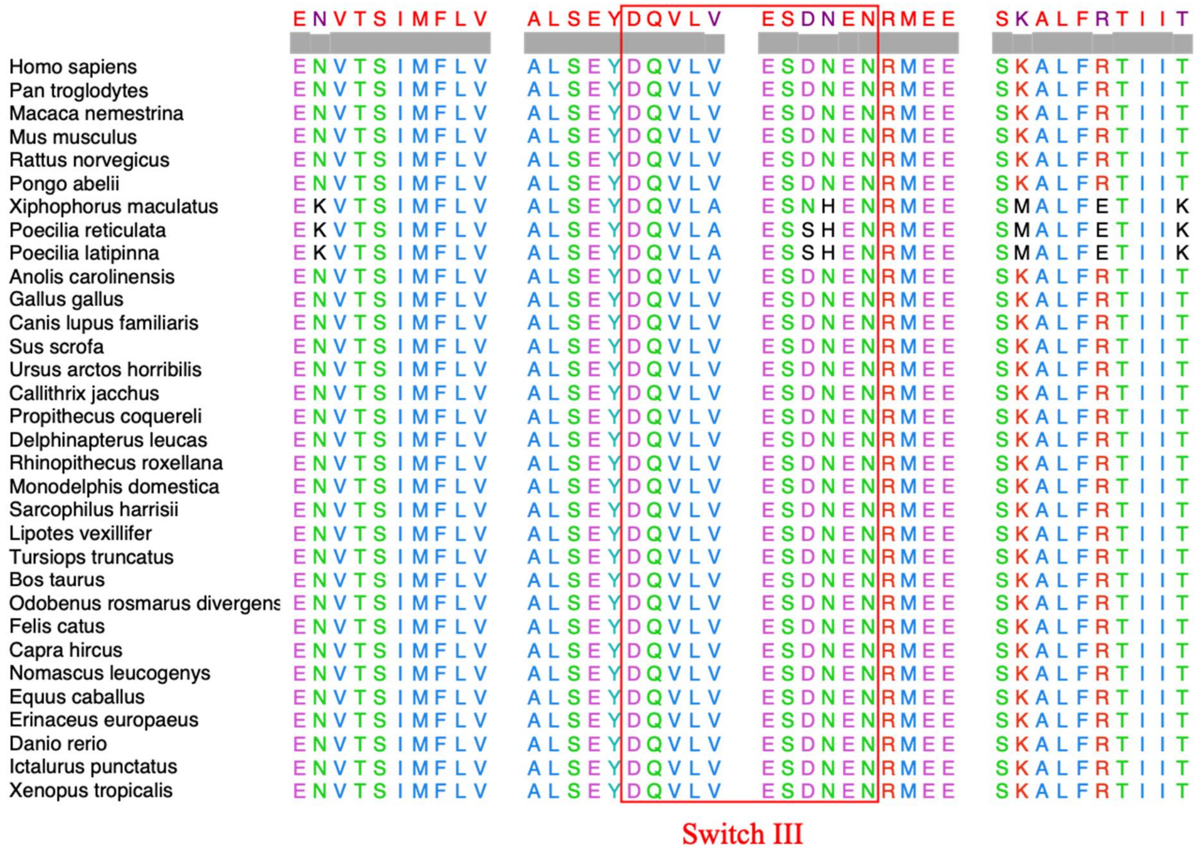

Figure S8. Sequence alignment of *GNAQ* orthologs centred on the Switch III loop (236-246 based on human sequence)

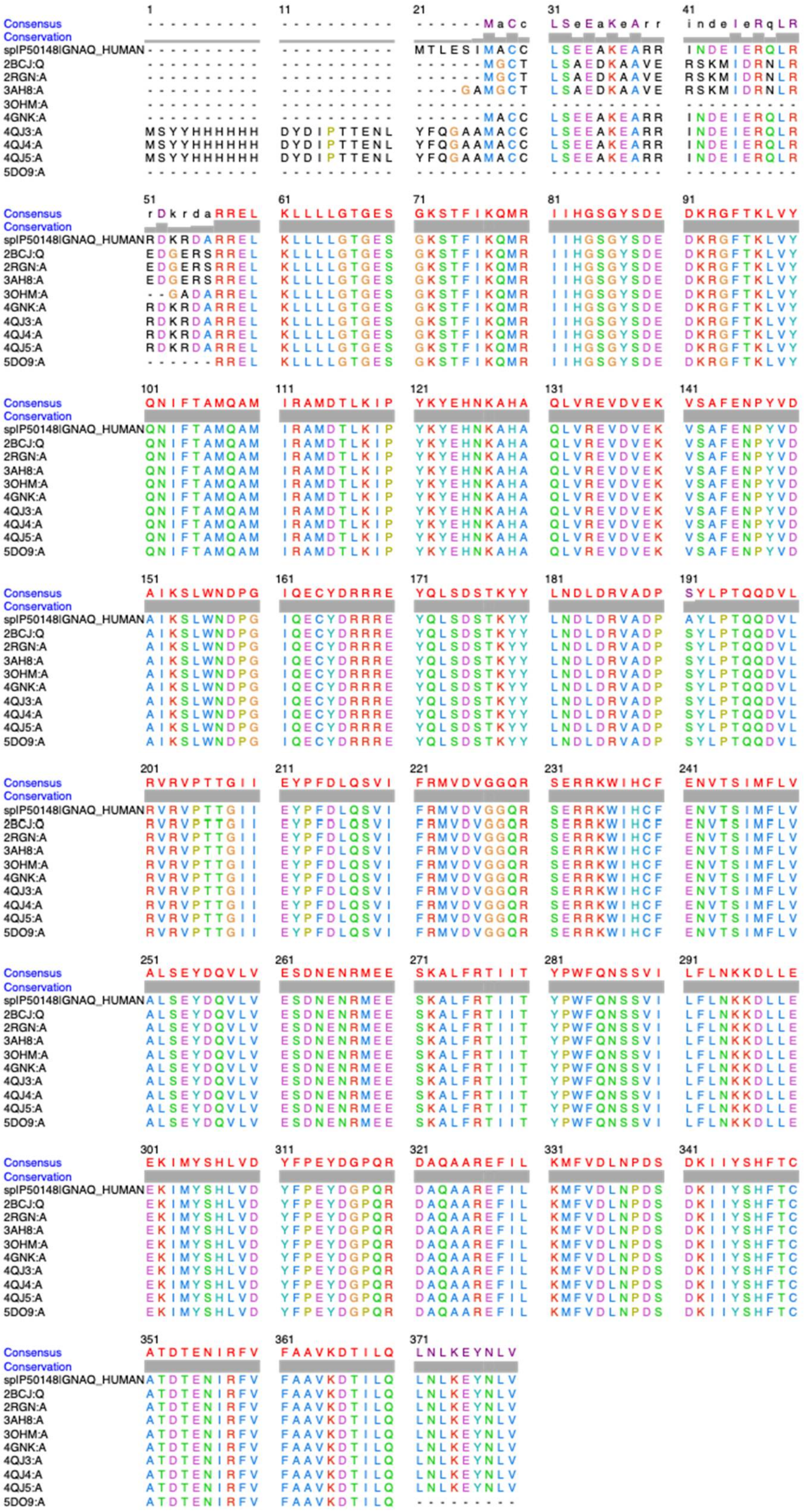

Figure S9. Sequence alignment of the PDB sequences of the chains of interest and the Human sequence of *GNAQ*, retrieved from UniProt

For our analysis, we decided to analyse experimental structures from *Mus musculus* Gα_q and *Mus Musculus x Rattus norvegicus* Gα_i/Gα_q chimera, as they share high sequence identities with human

$G\alpha_q$. Structures were retrieved from the PDB [2]: 3ah8 [3] (sole inactive conformation), 2bcj [4], 2rgn [4], 3ohm [5], 4gnk [6], 4qj3 [7], 4qj4 [7], 4qj5 [7], 5do9 [8]. Two other structures of *Mus musculus* $G\alpha_q$ protein are available in the PDB (i.e., 4ekd, 4ekc). As they contain mutations, we chose not to include them in our study. A description of the structure information is presented in Table .

Table S2. 3D structures information. A is for active conformation and I for inactive conformation. * Sequence identities between the human and the chain sequences were calculated using Chimera.

PDB_id	Organism	Protein	Chain	Identity with human $G\alpha_q$*	Resolution (Å)	R-value Free	R-value work	pH	Temperature (K)
3ah8 (I)	*Rattus norvegicus*, *Mus musculus* (chimeric)	$G\alpha_i/G\alpha_q$	A	94.1%	2.9	0.315	0.259	5.1	293
2rgn (A)	*Rattus norvegicus*, *Mus musculus* (chimeric)	$G\alpha_i/G\alpha_q$	A	94.6%	3.5	0.299	0.243	6.5	277
2bcj (A)	*Rattus norvegicus*, *Mus musculus* (chimeric)	$G\alpha_i/G\alpha_q$	Q	94.6%	3.06	0.292	0.243	6.5	277
4gnk (A)	*Mus musculus*	$G\alpha_q$	A	99.7%	4.00	0.255	0.214	6.75	277.15
5do9 (A)	*Mus musculus*	$G\alpha_q$	A	99.7%	2.60	0.226	0.178	5.5	277.15
3ohm (A)	*Mus musculus*	$G\alpha_q$	A	99.1%	2.70	0.275	0.204	6	291
4qj3 (A)	*Mus musculus*	$G\alpha_q$	A	98.1%	3.00	0.240	0.195	6	277.15
4qj4 (A)	*Mus musculus*	$G\alpha_q$	A	98.1%	3.30	0.267	0.208	6.5	277.15
4qj5 (A)	*Mus musculus*	$G\alpha_q$	A	98.1%	3.41	0.277	0.212	6.25	277.15

All structures were obtained with high resolution and the chains of interest have high sequence identities with the human $G\alpha_q$. The pH range of crystals preparation is comprised between 5.1 for 3ah8 and 6.25 for 5do9, and the temperature ranges from 277K for 2grn and 2bcj, to 291K for 3ah8.

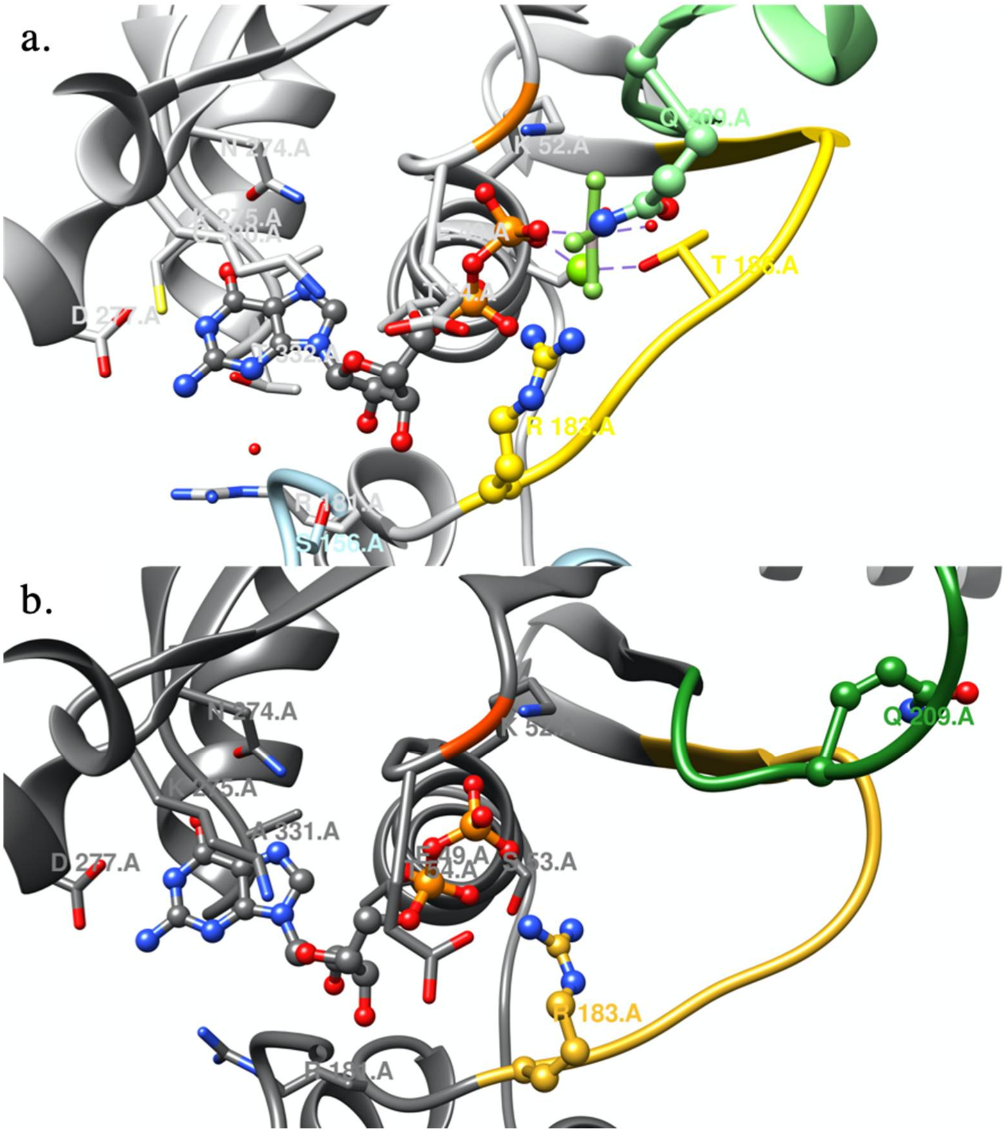

Figure S10. Residues in contact with ligands. (**a**). Active conformation crystallized in the presence of GDP, AlF_4 and Mg^{2+}(PDB ID3ohm); (**b**) Inactive conformation crystallized in the presence of GDP (PDB ID3ah8). Hotspots Arg183 and Gln209 and GDP and AlF_4 are in ball&stick, Mg^{2+} is in sphere, and the residues in contact with ligands are in sticks. SW-I (183-192) is in gold and SW-II (206-222 in green. SW-III is cut from the pictures to make them readable.

Table S3. FoldX energy analysis. Gibbs energies (ΔG) of each complex calculated by FoldX in kcal/mol.

PDB-ID	conformation	ΔG – WT (kcal/mol)	ΔG – MUT (kcal/mol)	ΔΔG (kcal/mol)
3ah8	Inactive	686.08	685.7	0.38
2rgn	Active	623.772	622.804	0.968
4gnk	Active	770.213	769.101	1.112
5do9	Active	107.297	106.156	1.141
3ohm	Active	787.41	785.7	1.71
4qj4	Active	458.572	456.77	1.802
4qj5	Active	651.392	649.573	1.819
2bcj	Active	697.385	695.083	2.302
4qj3	Active	334.318	331.149	3.169

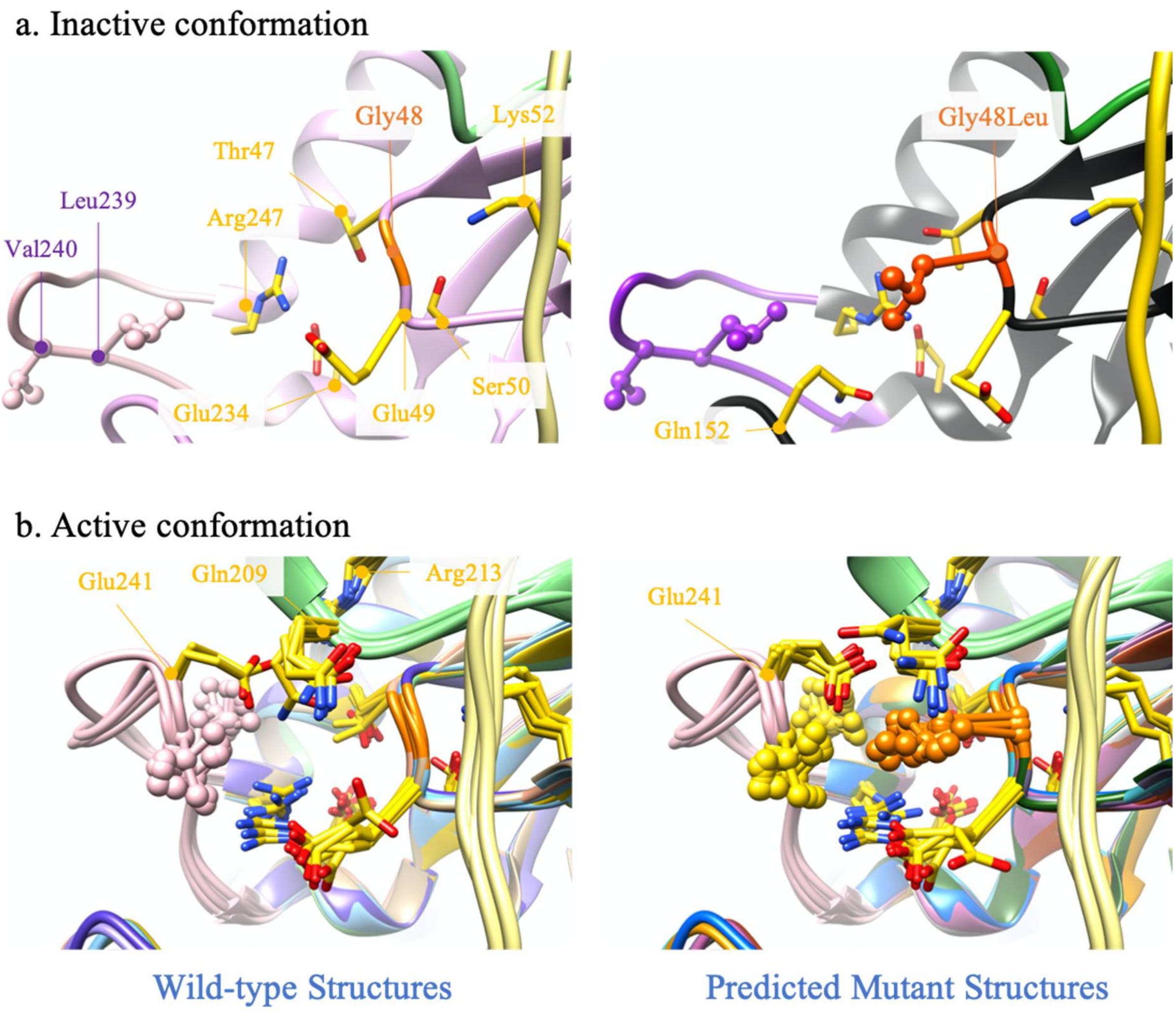

Figure S11. Wild-type and Mutated residue's neighbors. (**a**). Inactive structures (3ah8); (**b**). Active structures (2bcj, 2rgn, 3ohm, 4gnk, 4qj3, 4qj4, 4qj5, 5do9). Gly48Leu, Leu239 and Val240 are in ball&stick, residues that are at a distance inferior to 5Å to position 48 are in golden sticks. SW-I, SW-II and SW-III are in yellow, green and pink, respectively. Position 48 is colored in orange. For clarity, residues that are already mentioned in (a)-wild-

type are not mentioned in the other pictures. Left pictures represent the wild-type structures and the right one the Gly48Leu predicted structures generate with FoldX.

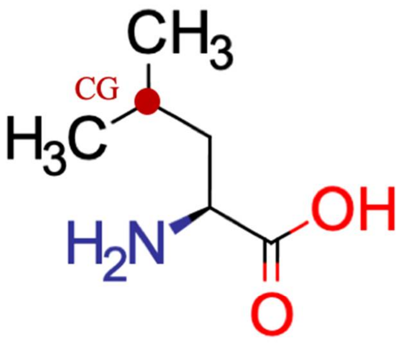

Leucine

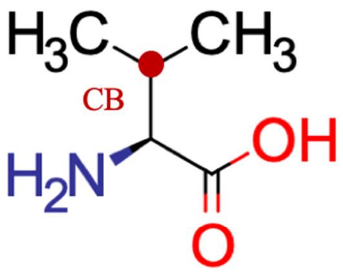

Valine

PDB-ID	Conformation	Distance (Å)	
		V240_CB-G48L_CG	L239_CG-G48L_CG
3ah8	Inactive	8.769	15.823
2rgn	Active	5.447	6.971
4gnk	Active	5.256	7.048
5do9	Active	5.44	6.858
3ohm	Active	5.234	6.274
4qj4	Active	5.315	6.037
4qj5	Active	5.393	7.178
2bcj	Active	5.435	6.626
4qj3	Active	5.274	6.481

Figure S12. Structural analysis. Distances measured between atoms L239 C and G48L C , and V240 C and G48L C .

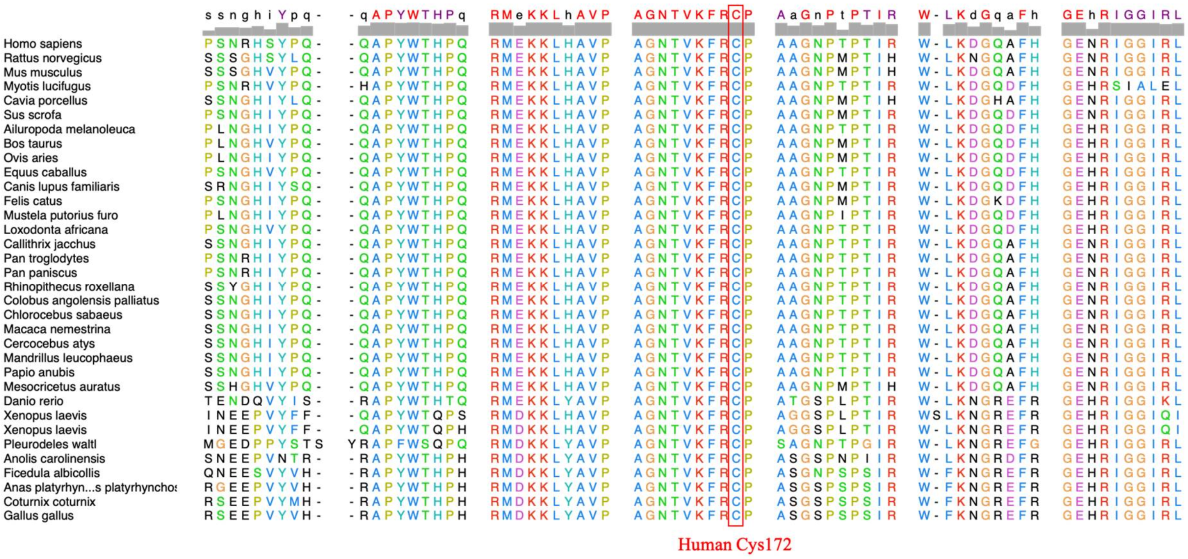

Figure S13. *FGFR4* Multiple sequence alignment centered on the Homo sapiens Cys172. Sequence retrieved from UniProt and alignment done with MUSCLE.

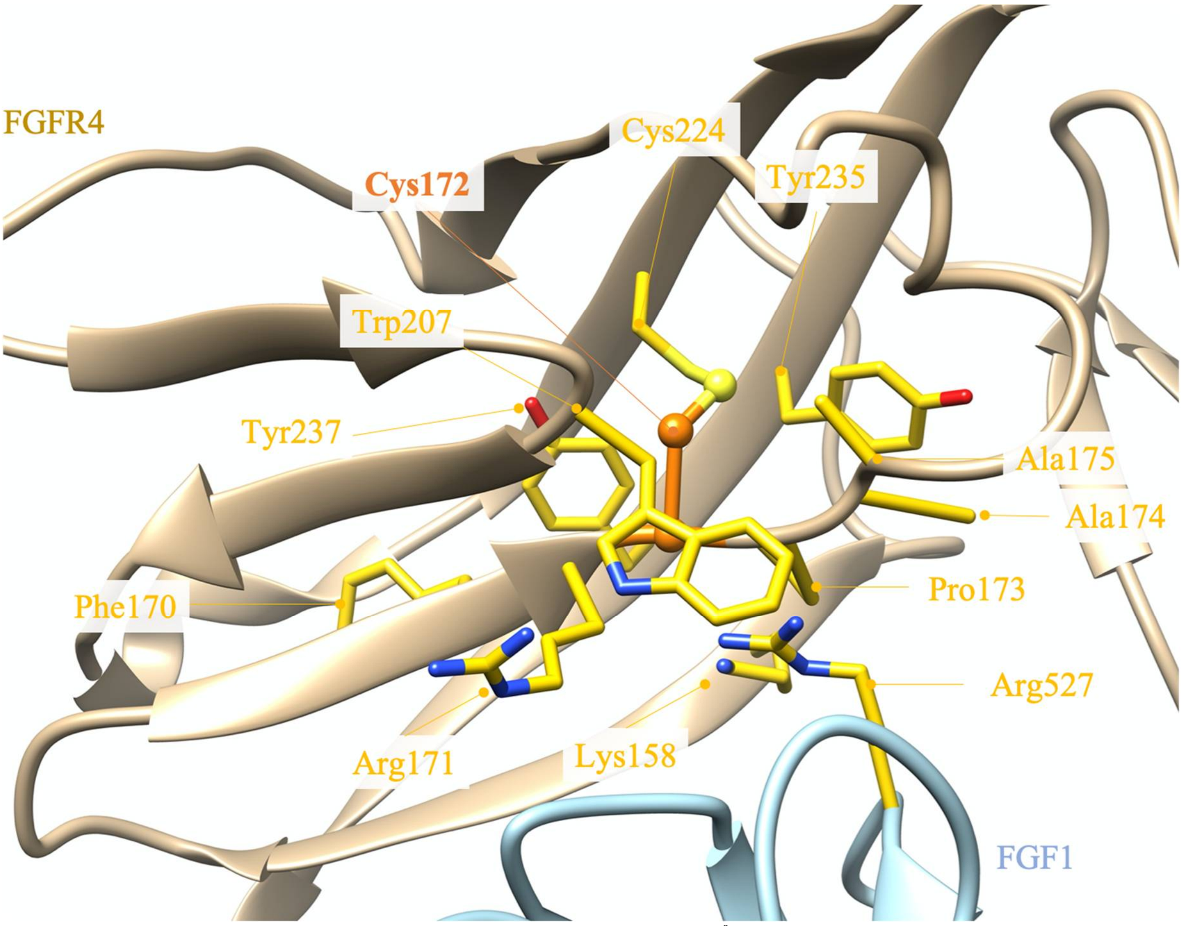

Figure S14. *FGFR4* p.Cys174 environment. Residues that are within 5Å from the *FGFR4* p.Cys174 are represented in golden sticks. Cys172 is in orange ball&stick. FGFR4 is represented in tan ribbon and FGF1 in light blue ribbon (PDB ID 1qct) [9]

Table S4. The results of the in-house NGS panel. BAP1 presented both with a mutation and a loss of one allele leading to a loss of heterozygosity (LOH)

Gene ID	Alterations	Allele frequency or level of CNV
GNAQ	c.142_143delinsTT (p.Gly48Leu)	41%
FGFR4	c.514T>G (p.Cys271Gly)	47%
BAP1	c.68-4_84delinsGA and LOH of 3p	82% (due to LOH)
NRAS	Deletion	1x copy left
3p	Loss	LOH
6q	Loss	LOH
6p	Gain	3x

References

[1] Potapov, V.; Cohen, M.; Schreiber, G. Assessing computational methods for predicting protein stability upon mutation: Good on average but not in the details. *Protein Eng. Des. Sel.* **2009**, *22*, 553–560. https://doi.org/10.1093/protein/gzp030.

[2] Moorhouse, M.; Barry, P. The Protein Databank. *Bioinforma. Biocomput. Perl*, **2005.** https://doi.org/10.1002/0470020571.ch10.

[3] Nishimura, A.; Kitano, K.; Takasaki, J.; Taniguchi, M.; Mizuno, N.; Tago, K.; et al. Structural basis for the specific inhibition of heterotrimeric G q protein by a small molecule. *Proc. Natl. Acad. Sci.* **2010**, *107*, 13666–13671. https://doi.org/10.1073/pnas.1003553107.

[4] Tesmer, V.M.; Kawano, T.; Shankaranarayanan, A.; Kozasa, T.; Tesmer, JJG. Snapshot of Activated G Proteins at the Membrane: The Gαq-GRK2-Gßγ Complex. *Science* (80-) **2005**.

[5] Waldo, G.L.; Ricks, T.K.; Hicks, S.N.; Cheever, M.L.; Kawano, T.; Tsuboi, K.; et al. Kinetic scaffolding mediated by a phospholipase C-β and Gq signaling complex. *Science* (80-) **2010**, https://doi.org/10.1126/science.1193438.

[6] Lyon, A.M.; Dutta, S.; Boguth, C.A.; Skiniotis, G.; Tesmer, JJG. Full-length Gαq-phospholipase C-β3 structure reveals interfaces of the C-terminal coiled-coil domain. *Nat. Struct. Mol. Biol.* **2013**, https://doi.org/10.1038/nsmb.2497.

[7] Lyon, A.M.; Tesmer, JJG. Structural insights into phospholipase C-β function. *Mol. Pharmacol.* **2013**, https://doi.org/10.1124/mol.113.087403.

[8] Taylor, V.G.; Bommarito, P.A., Tesmer, JJG. Structure of the Regulator of G Protein Signaling 8 (RGS8)-Gα q Complex. *J. Biol. Chem.* **2016**, https://doi.org/10.1074/jbc.m115.712075.

[9] Huhtala, M.T.; Pentikainen, O.T.; Johnson, M.S. A dimeric ternary complex of FGFR1, heparin and FGF-1 leads to an "electrostatic sandwich" model for heparin binding. *Structure* **1999**, *7*, 699–709, https://doi.org/10.1016/S0969-2126(99)80129-4.
